# Supplementary material for: RAD-TGTs: high-throughput measurement of cellular mechanotype via rupture and delivery of DNA tension probes
Source: Nat Commun. 2023 Apr 28;14:2468. doi: 10.1038/s41467-023-38157-6 (PMC10147940; doi:10.1038/s41467-023-38157-6)
Supplement: Supplementary file 1 — Supplementary Information [file 41467_2023_38157_MOESM1_ESM.pdf]

Supplementary Information

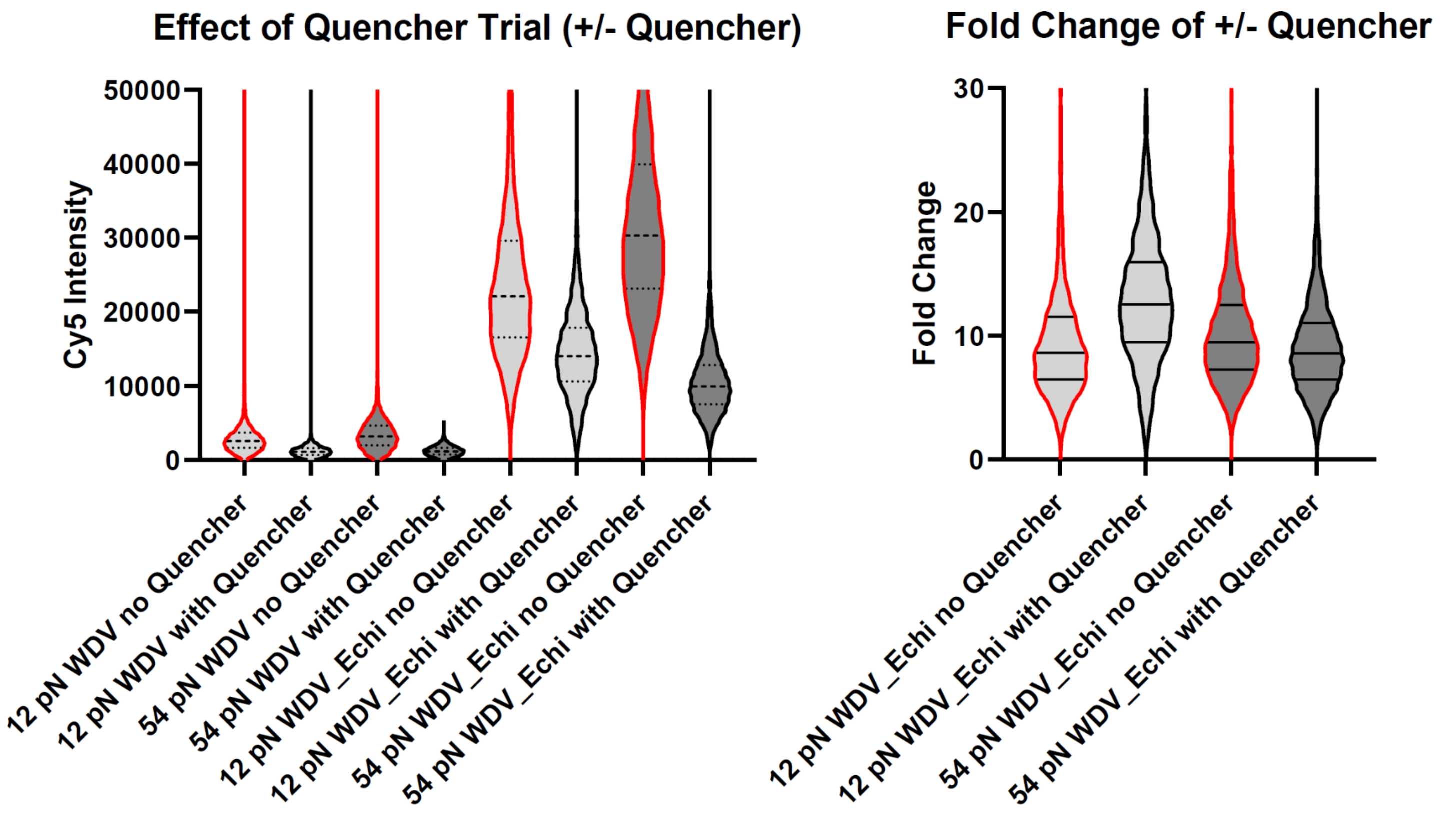

Supplementary Fig. 1. **RAD-TGT Readout With or Without Quencher on Bottom Strand.** U251 cells were incubated on 12 (light grey) or 54 pN (dark grey) RAD-TGT coated surfaces. Each condition was performed with (black outline) and without a quencher (red outline) present on the bottom strand. Cy5 intensity was measured for each condition and associated fold change of CY5 fluorescence relative to WDV only was calculated and graphed. Horizontal lines represent the median and interquartile range.

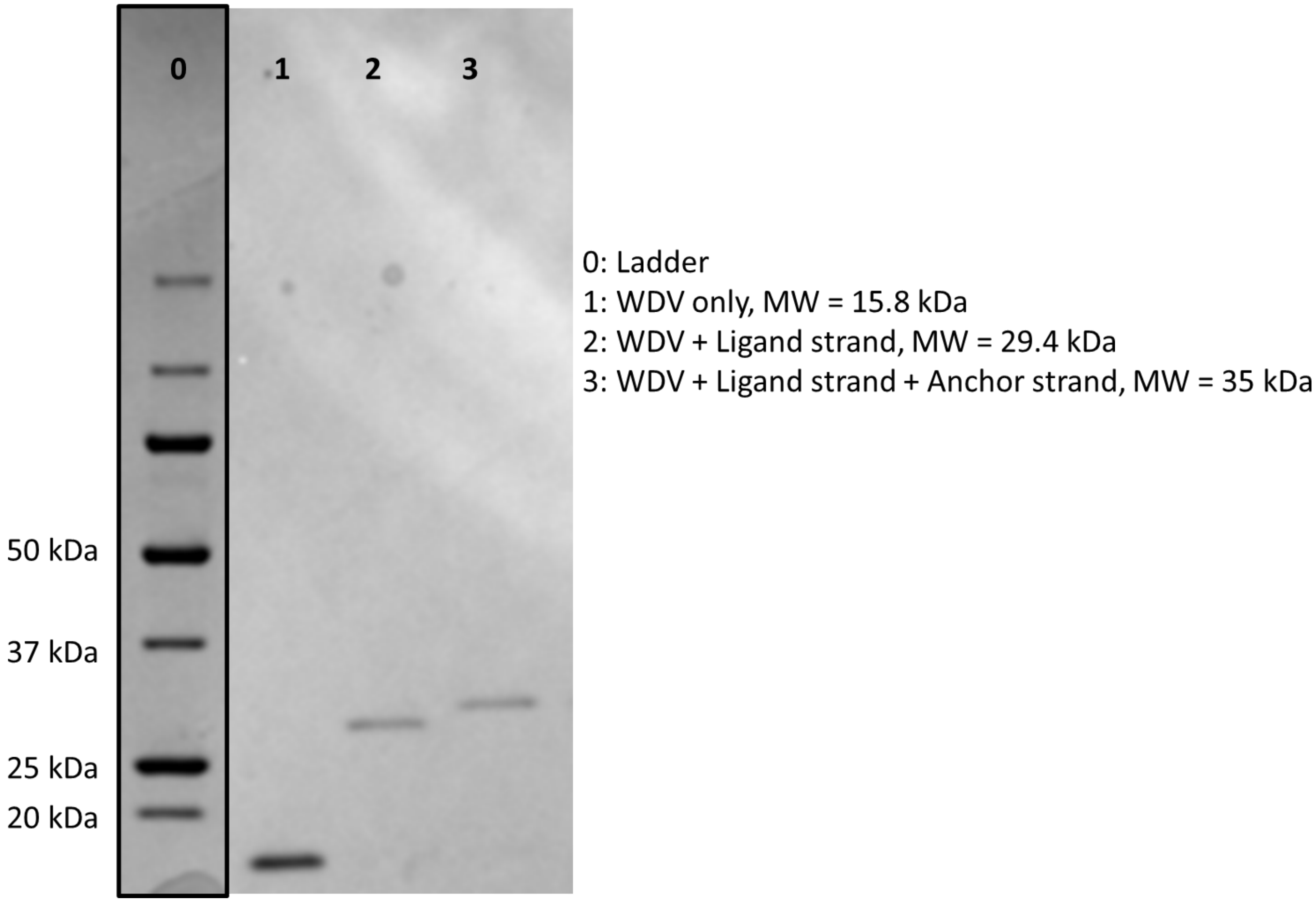

Supplementary Fig. 2. **HUH reacting with ssDNA and duplex DNA captured via SDS poly acrylamide gel electrophoresis.** RAD-TGT components were reacted and analyzed in a non-denaturing polyacrylamide gel. The reaction for lane 1 only contained WDV, lane 2 contained the ligand strand from RAD-TGT and excess WDV, lane 3 contained both ligand and anchor strands from the RAD-TGT annealed together and excess WDV. Lane 1 contains a singular band representing WDV while lanes 2 and 3 contain two bands, WDV and a WDV-DNA band below that. The new bands in lanes 2 and 3 is the WDV reacting with the oligos present, the oligos carry a negative charge so when bound to WDV migration distance increases on a native gel. The band in lane 3 is slightly shifted up relative to the band in lane 2, this is because the annealed duplex increases the mass of the complex resulting in slower migration. From this gel it is evident that the WDV can react with the duplex DNA present in RAD-TGTs. Source data are provided as a Source Data file.

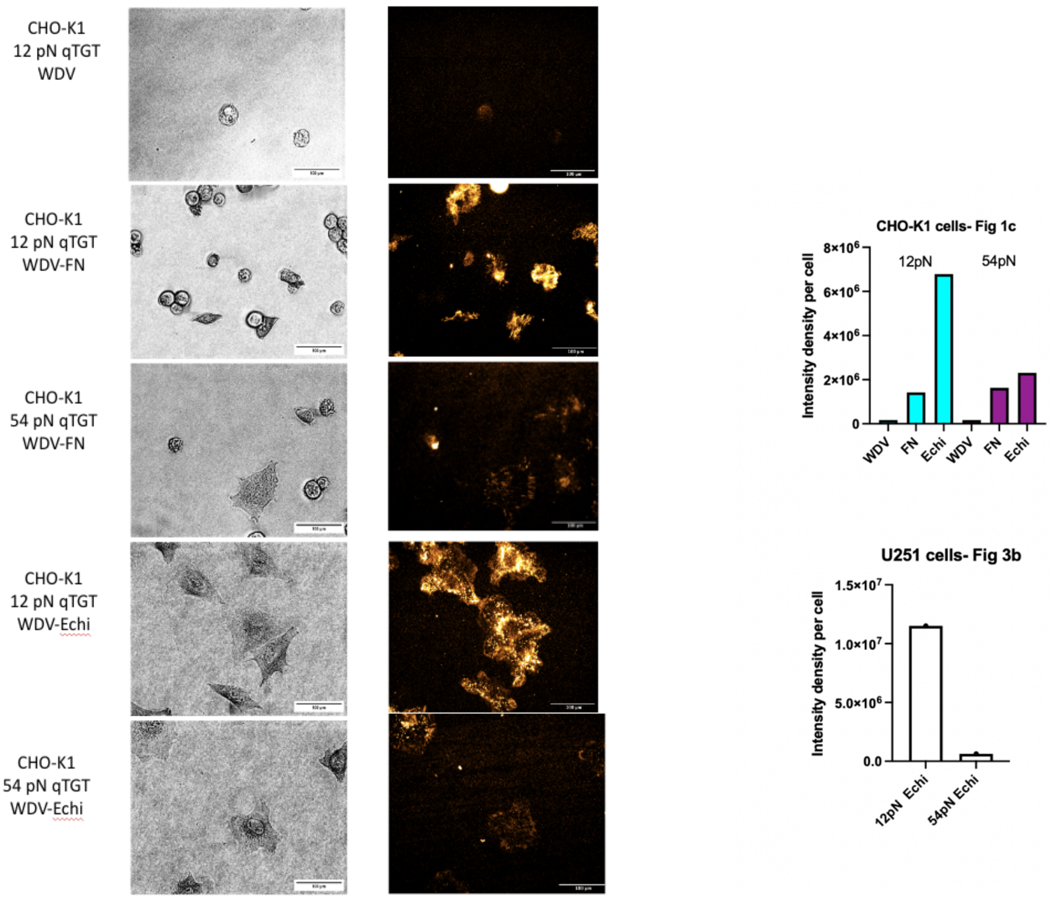

Supplementary Fig. 3. **Supplementary Fig. 3**. (left) Brightfield and wider view images corresponding to Fig 1c. (right) Quantification of fluorescence intensity density per cell in Fig 1c and 3b. A ROI corresponding to a cell was chosen in imageJ, the intensity density was measured across the cell area. 4-10 cells were analyzed per condition.

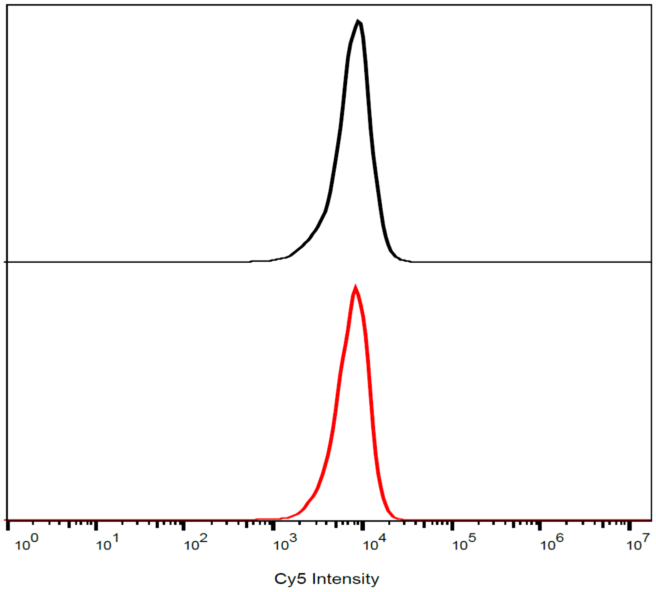

	Condition	Median Cy5 Intensity	Count
☐	No Nuclease Treatment	8038	4083
☐	Nuclease Treated	7781	3822

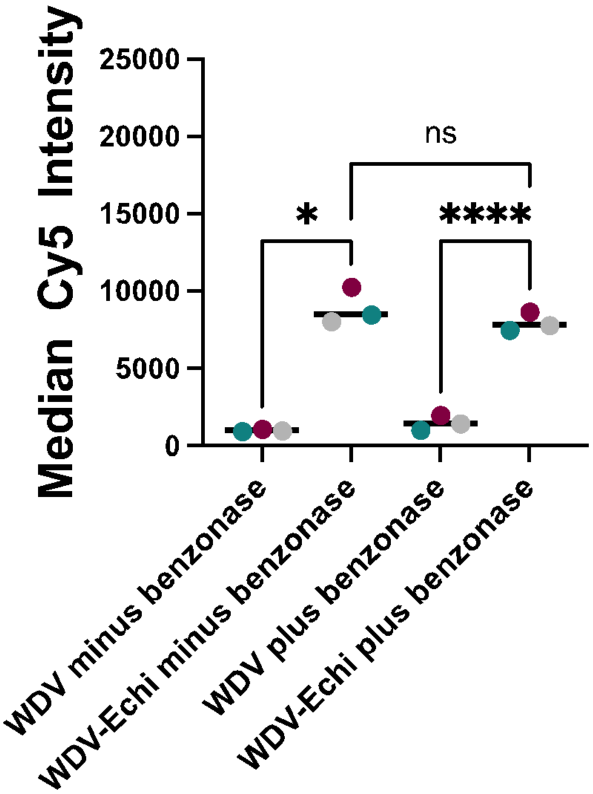

Supplementary Fig. 4. Nuclease treatment of U251 cells. U251 cells were plated on Cy5- labeled RAD-TGTs conjugated to WDV-Echistatin. Following a 90 minute incubation cells were trypsinized and resuspended in a modified resuspension buffer that did not contain any EDTA (1% BSA in PBS). 2 mM $MgCl_2$ and 0.5 U/µL benzonase (Milipore, E1014) were added to the solution; for no nuclease treatment conditions benzonase buffer (50% glycerol containing 20 mM Tris HCl, pH 8.0, 2 mM MgCl2, and 20 mM NaCl) was used in lieu of benzonase. Solutions were incubated at 37 °C for 10 minutes followed by addition of 10 mM EDTA to quench the reaction. Cells were then analyzed via flowcytometry as previously described. (left) representative histograms of nuclease and no nuclease treated cells. (right) Median Cy5 intensities of three biological replicates of WDV and WDV-Echi TGTs with and without benzonase. Statistical significance was calculated by one-way ANOVA in Graphpad prism. *p=0.0162; **** p<0.0001.

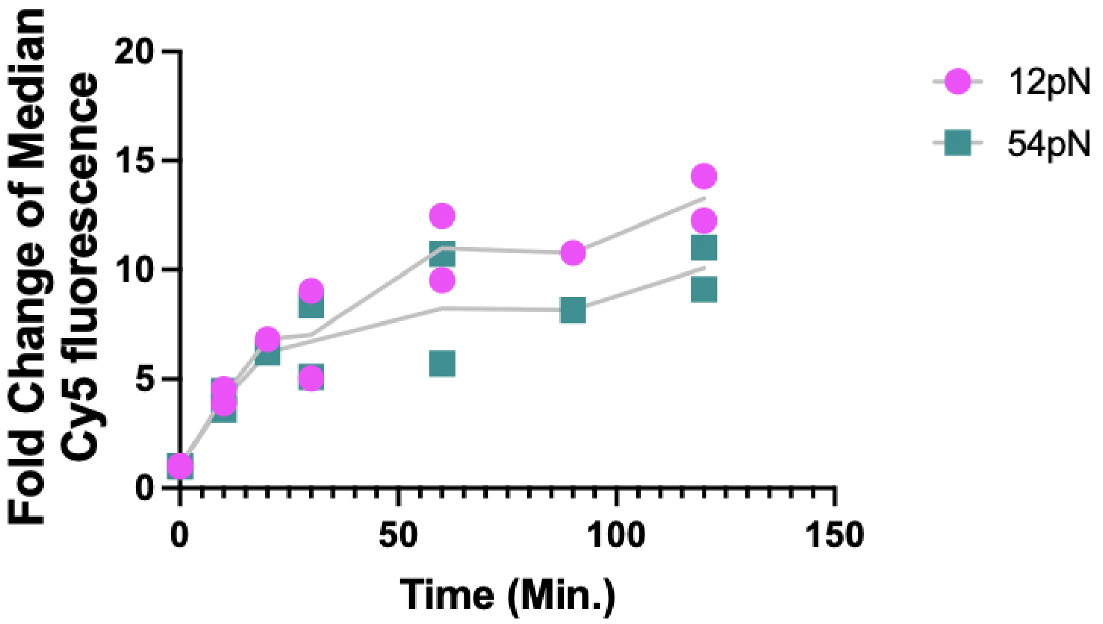

Supplementary Fig. 5. Incubation time on RAD-TGTs increases fluorescence intensity. U251 cells were incubated on echistatin conjugated 12 and 54 pN RAD-TGT surfaces. Cells were incubated for varying time points from 10 minutes to 120 minutes, once desired time was reached cells were dissociated and immediately analyzed via flow cytometry. The median fluorescence at each time point was normalized for the WDV alone median fluorescence. The graph shows two biological replicates, except at 90 minutes. The line represents the average fold change of the two experiments.

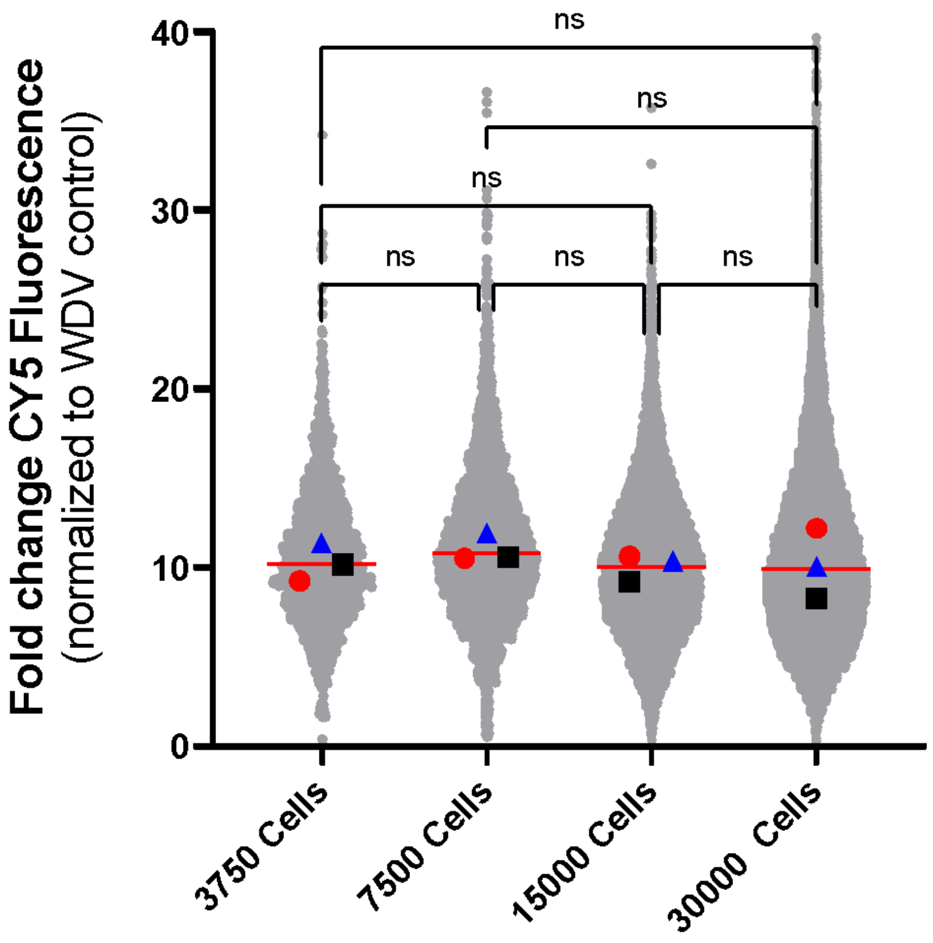

Supplementary Fig. 6. Impact of seeded cell count on fluorescent intensity. A titration of U251 cells (from cell count 3750 to 30000) were plated on WDV or WDV-Echi conjugated to 12 pN RAD-TGT wells and incubated for 90 minutes in 96 well matek glass bottom plates. Grey dots represent Cy5 fluorescence of each cell combining 3 biological replicates. Fold change relative to WDV MFI was calculated for each density. Colored symbols represent the median fold change of fluorescence per biological replicate. Red horizontal line is at the median for each condition. Statistics were performed using a one way ANOVA of the medians of three biological replicates. N.s $p>0.05$. Source data are provided as a Source Data file.

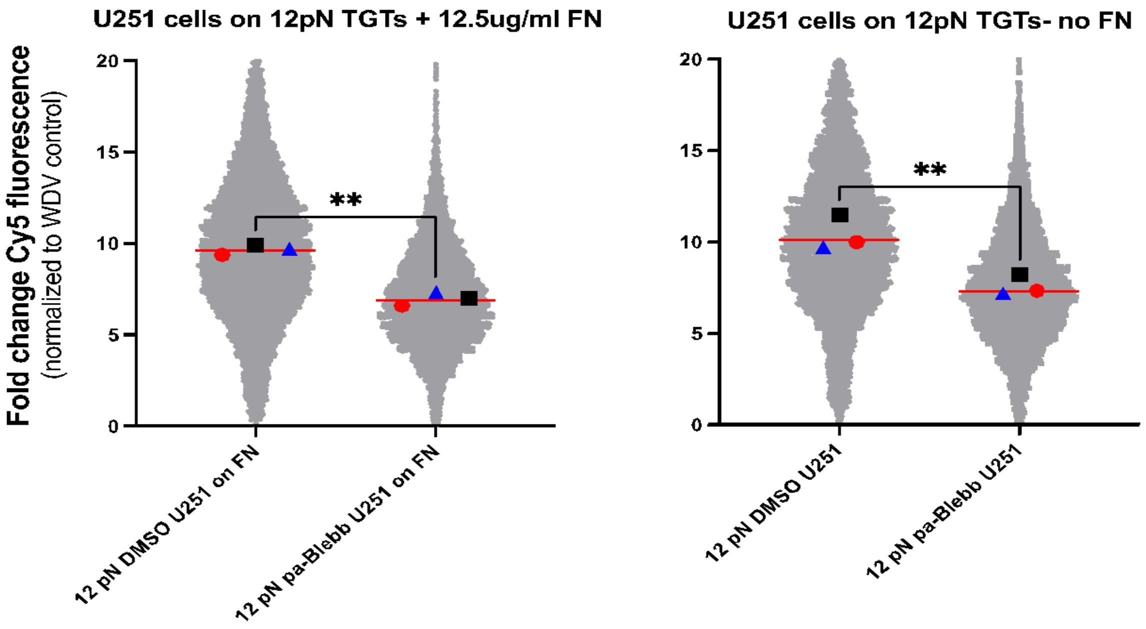

Supplementary Fig. 7. RAD-TGTs Function with and without fibronectin present. 12 pN RAD-TGTs were plated on both clean glass surfaces and surfaces that were coated with 12.5 ug/ml fibronectin. U251 cells were plated on both surfaces and with either WDV or WDV-Echi RAD-TGTs and were treated with para-amino-Blebbistatin or DMSO as a vehicle control. Grey dots represent Cy5 fluorescence of each cell combining all 3 biological replicates. Colored symbols represent the median fold change of fluorescence of WDV-Echi compared to WDV alone per biological replicate. Statistics were calculated by two tailed paired T-test of median fold change values. **p= 0.0041 (+FN), 0.006 (-FN) The trend between cells on glass or fibronectin was preserved. Red horizontal line is at the median for each condition. Source data are provided as a Source Data file.

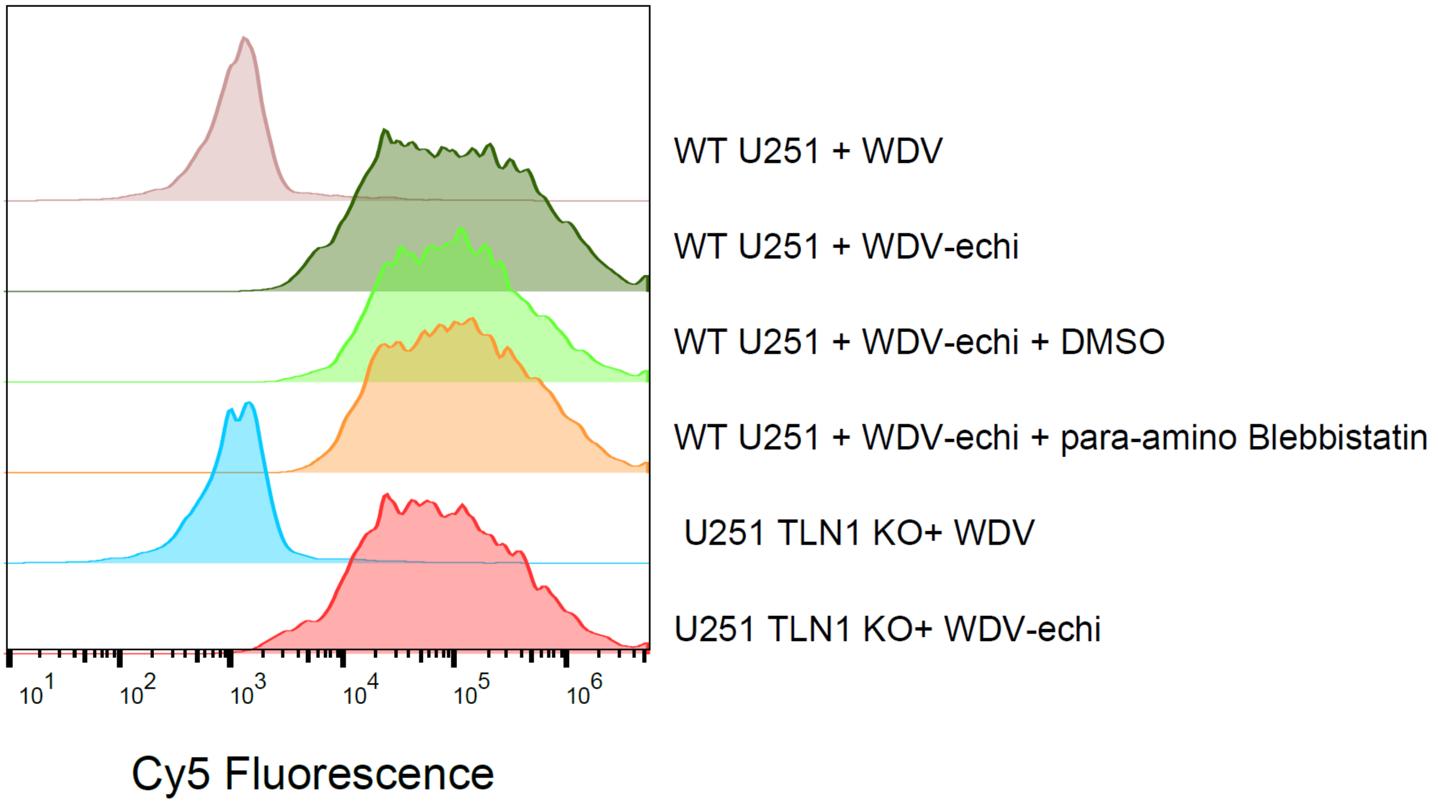

Supplementary Fig. 8. Representative histograms of U251 cells on polystyrene plates. Please note we did NOT use polystyrene plates in the manuscript- these data demonstrate why. U251 cells were incubated on an echistatin conjugated RAD-TGT surface as described in the main text but rather than glass plates neutravidin coated polystyrene plates (Thermo Fisher, 15129) were used so no biotinylated-BSA or neutravidin was added to the plate. Surfaces contained either no igand WDV conjugated RAD-TGTs or echistatin conjugated RAD-TGTs present. Wild type U251 cells were tested with and without para-amino-blebbistatin treatment and U251 TLN1 KO cells were also measured. The vertical line is representative of the median Cy5 intensity for WDV-Echi. The distribution of events is broad relative to experiments on glass plates and a distinct tail forms in the presence of echistatin. We attributed these results to non-specific binding promoted by the readily adhering polystyrene causing uneven distribution of RAD-TGTs and fluorescently labeled oligos. Source data are provided as a Source Data file.

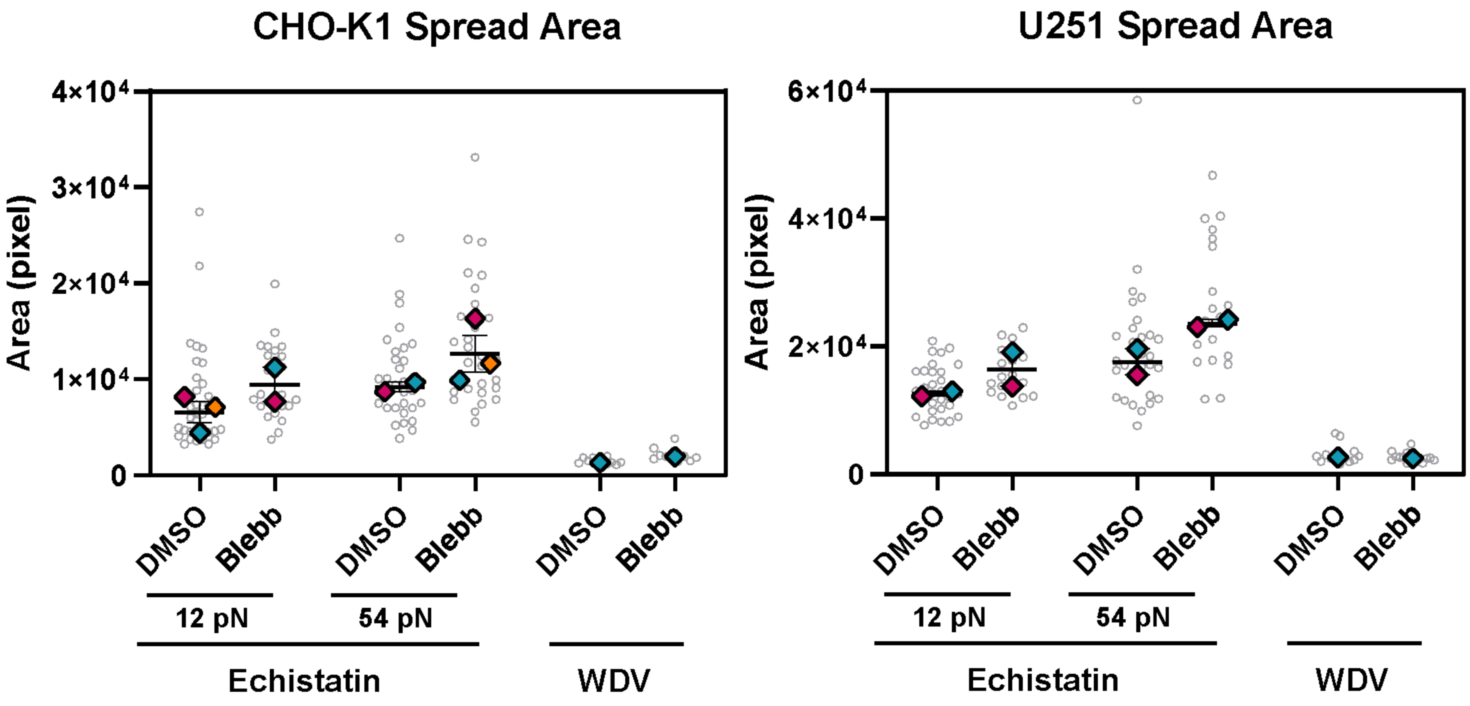

Supplementary Fig. 9. RAD-TGT Signal is Not Directly Related to Spread Area. Within the main text (fig. 2) it was observed that para-amino-Blebbistatin (Blebb) significantly decreases RAD-TGT signal relative to a DMSO control. Images of the cells before RAD-TGT readout were captured and the area (pixels) was then measured using FIJI. This was performed on both 12 and 54 pN RAD-TGTs using both WDV-Echi and WDV treated with a vehicle control (DMSO) or Blebb. Each colored point represents median cell area per image, horizontal lines and accompanying error bars represent the mean and SEM, each circle represents an individual cell measured. At least 8 cells were measured per WDV-echi or WDV-FN condition and at least 3 cells were measured per WDV only conditions. Source data are provided as a Source Data file.

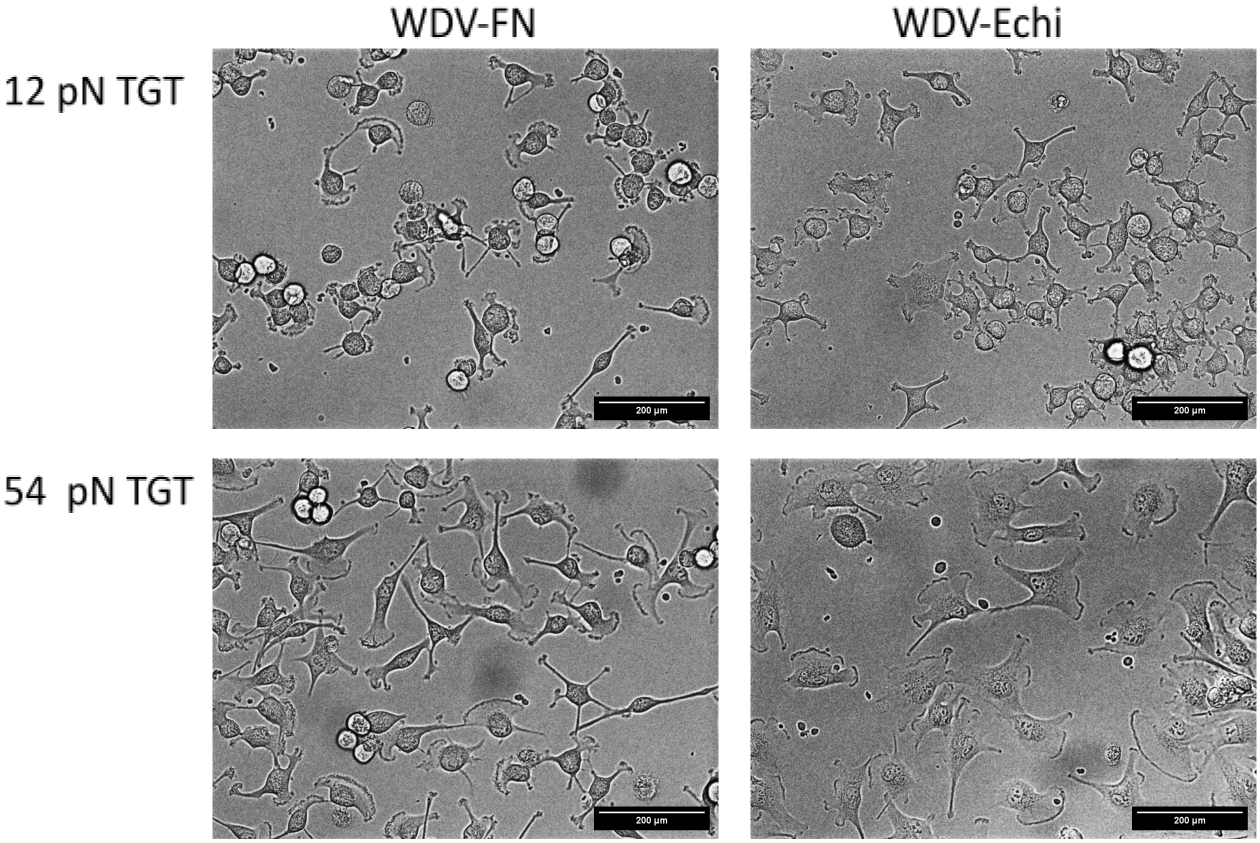

Supplementary Fig. 10. Brightfield images of U251 on different ligand-conjugated RAD-TGTs

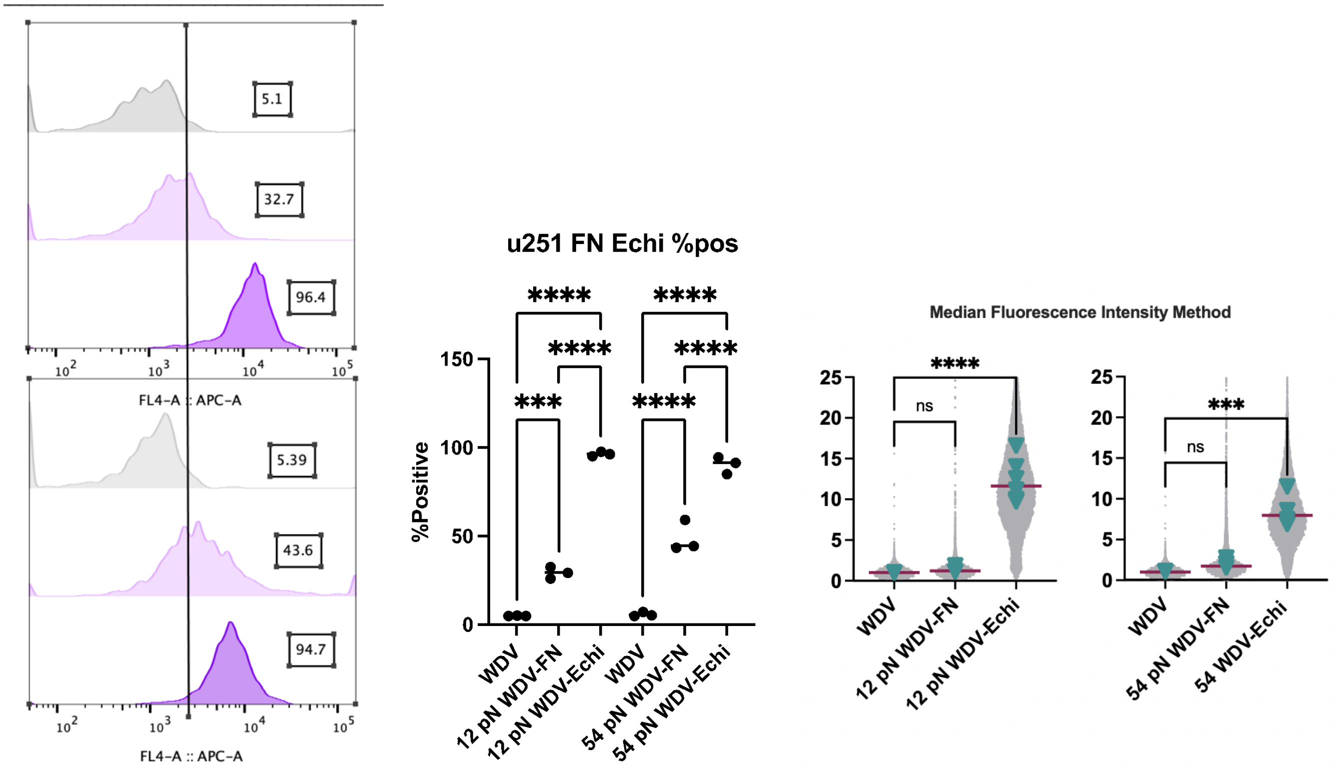

Supplementary Fig. 11. Comparing percent positive versus median fluorescence methods of analyzing data for data presented in Fig2A. The median and SuperPlot method was described for Fig 1, each symbol is the median fold change per replicate and the horizontal red line is set at the median value of the replicates. The horizontal red line is at the median fold change for all cells analyzed. n = 8 or 5 independent experiments for 12 pN and 54 pN graphs respectively. ***p = 0.0002, ****p < 0.0001, ns p>0.05 For the percent positive method, a gate is drawn such that the negative control population is ~95% below the gate. The percent positive cells above the set gate is the percent positive. For fold change SuperPlots, the red horizontal line is at the median for each condition. . Statistics were performed using a one way ANOVA of the medians of three biological replicates.***p=0.0002; ****p<0.0001. Source data are provided as a Source Data file.

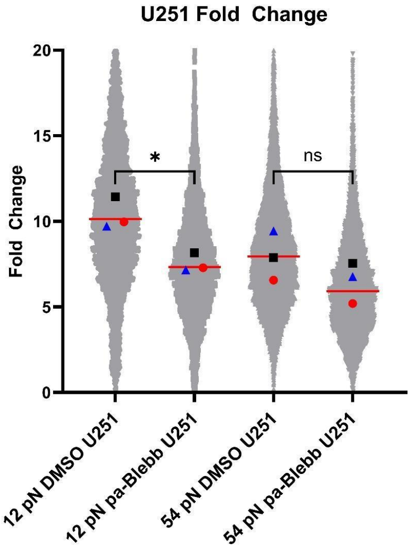

Supplementary Fig. 12. Blebbistatin treatment reduces RAD-TGT rupture by U251 cells. Fold change in CY5 fluorescence normalized to median fluorescence of WDV alone. Grey points are all fluorescent points from three biological replicates, while symbols are median of each data set. Statistics were calculated by one way anova of median fold change values of biological triplicates. ns p>0.05, * p=0.0151. Red horizontal line is at the median for each condition. Source data are provided as a Source Data file.

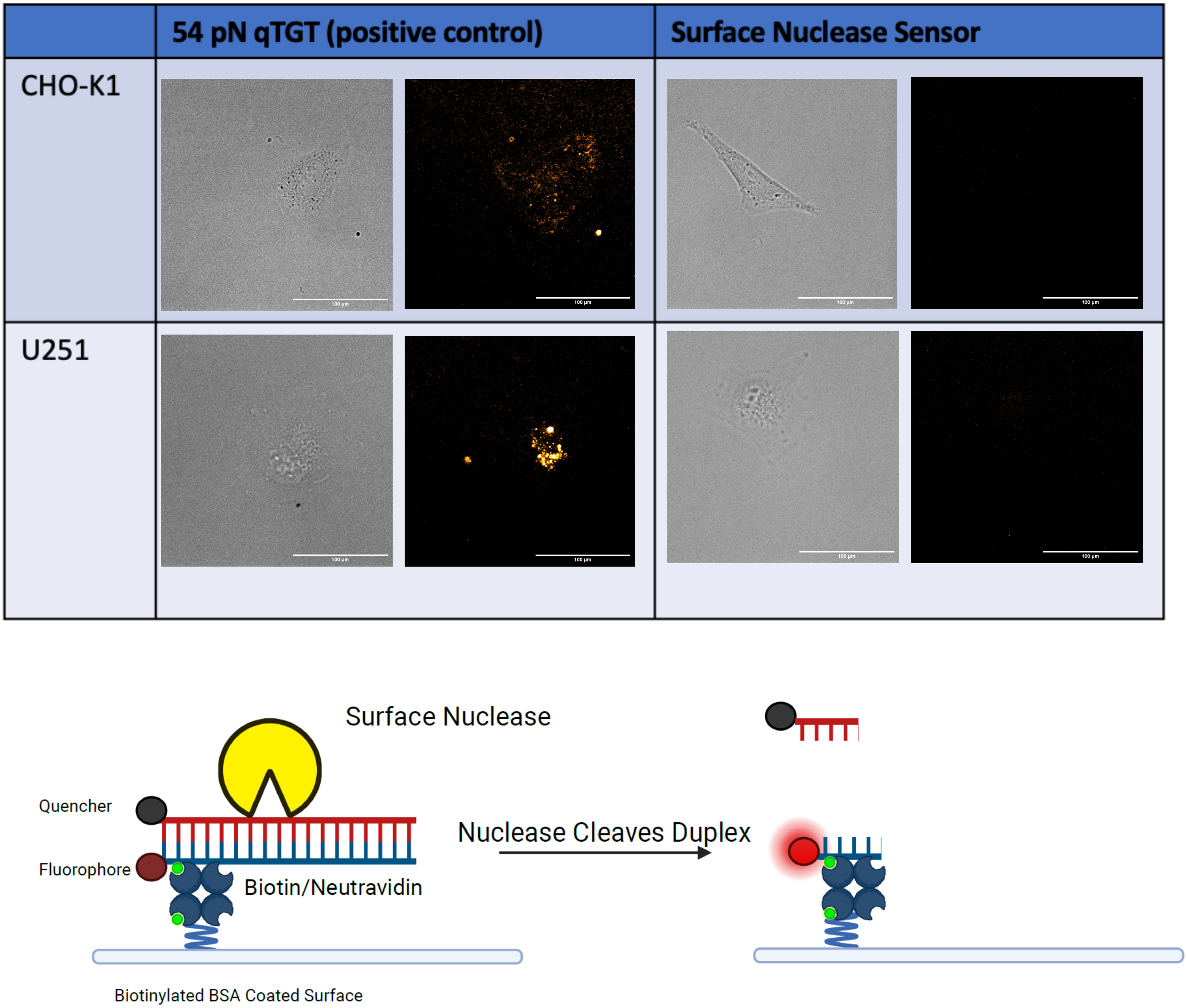

Supplementary Fig. 13. U251 have little surface nuclease activity. Both U251 and CHO-K1 cells were plated onto 54 pN qTGT surfaces that also contained fibronectin to allow for proper adhesion. The positive controls contained WDV-Echi so that one may visualize the normal rupture intensity. The surface nuclease sensor was similar in design but only had WDV present so there was no ligand. Both brightfield (left) and fluorescent images (right) were captured to see overall shape of the cell and any gain of fluorescence from duplex dissociation from either force or nuclease activity. Below is a cartoon of how surface nucleases sensors function gaining ,fluorescence if a nuclease is encountered without the need of a ligand. Cartoon created with BioRender.com.

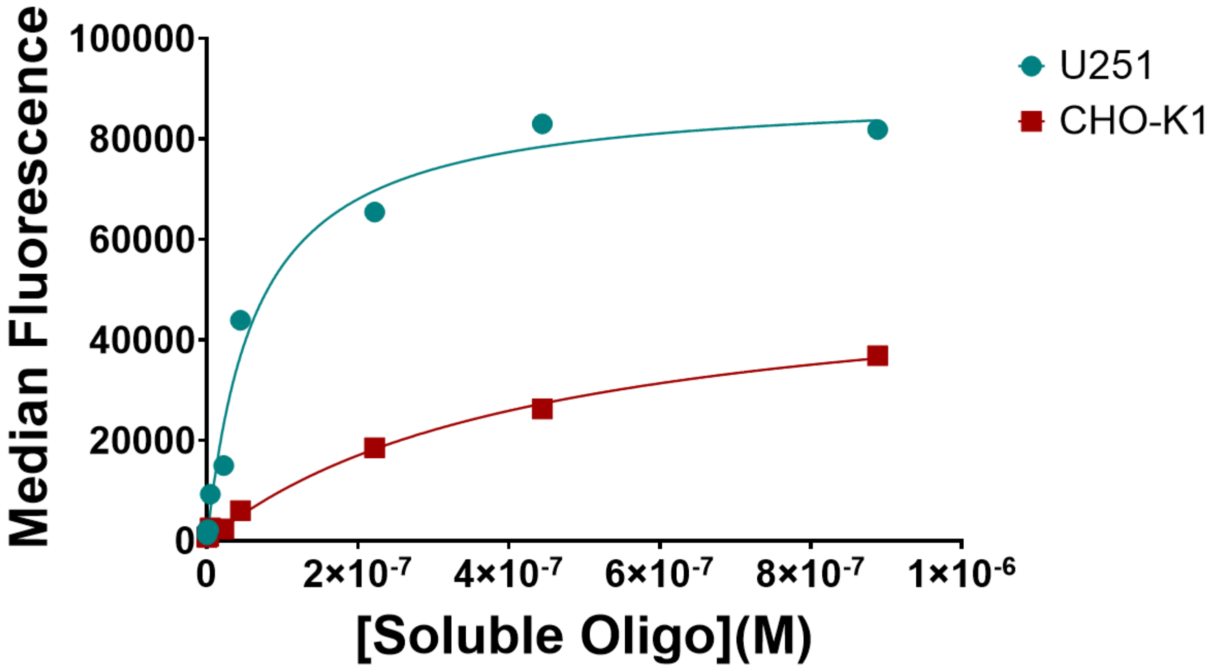

Supplementary Fig. 14. Soluble RAD-TGT Titration Highlights Differences in Integrin Composition of Between Cell Lines. 15,000 U251 and CHO-K1 cells were plated on fibronectin surfaces and allowed to adhere. Soluble RAD-TGTs composed of WDV-Echi conjugated to fluorescently labeled ligand strand annealed to the non-anchor strand was added into the wells at varying concentrations ranging from 0 to 0.89 μM. The total pmol range of oligo was from 0 to 160 pmol, the standard RAD-TGT experiment in the main text contained 80 pmol oligo assuming all oligo properly adhered, thus this is representative of experimental conditions. From this it is evident that U251 cells internalize the soluble RAD-TGT greater than the CHO-K1 cells. Source data are provided as a Source Data file.

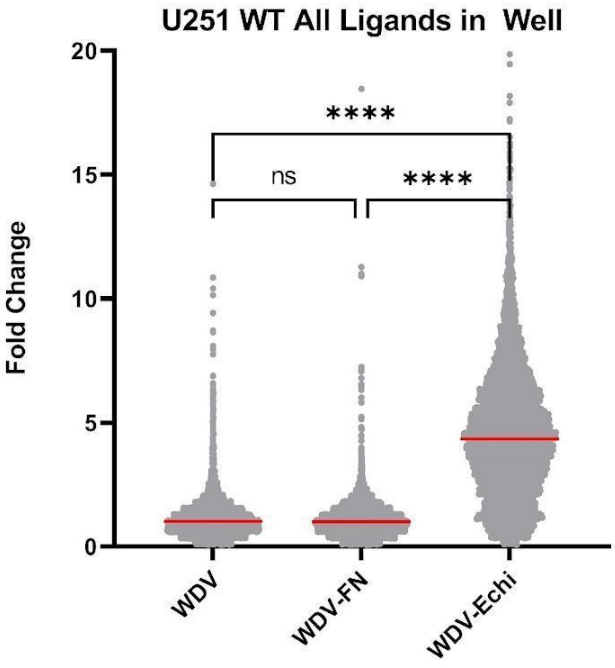

Supplementary Fig. 15. Flow Cytometry Data at 1/3 Ligand Concentration that Corresponds to Sequencing Data. RAD-TGTs with unique barcodes were conjugated to WDV, WDV-FN, and WDV-Echi and plated in a 96 well plate. Cells were plated, dissociated, and analyzed by flow cytometry while a duplicate sample was sent for sequencing. Statistics were performed using a one way ANOVA of all cells collected per condition. n.s $p > 0.05$, ****$p < 0.0001$. Red horizontal line is at the median for each condition. Source data are provided as a Source Data file.

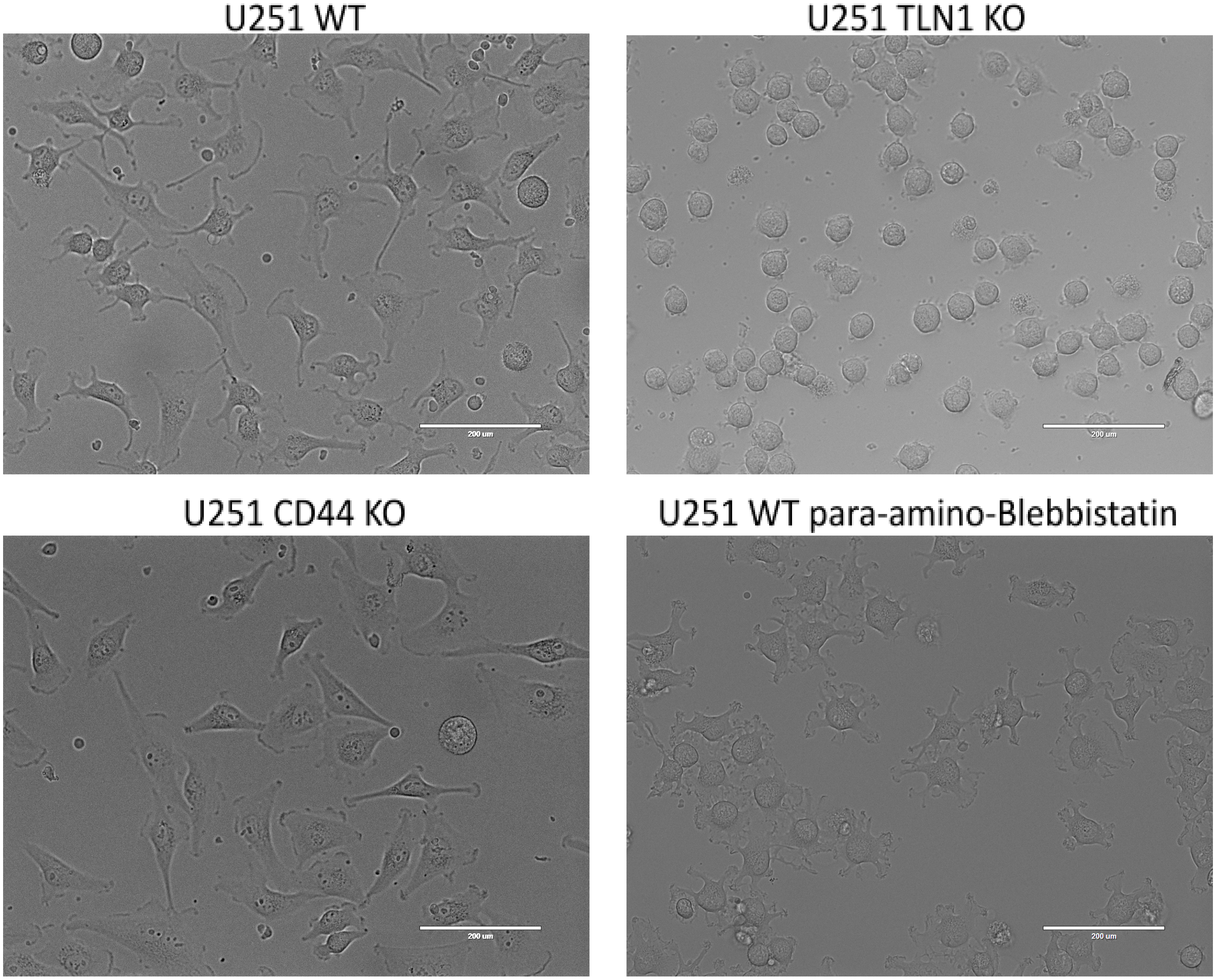

Supplementary Fig. 16. **Representative Images of U251 Cells on RAD-TGT surface**. All U251 cells and variants were plated on surfaces containing echistatin conjugated RAD-TGTs and allowed to incubate for 90 minutes. In total 4 conditions are shown here, U251 WT, U251 TLN1 KO, U251 CD44 KO, and U251 treated with 50 µM para-amino-Blebbistatin.

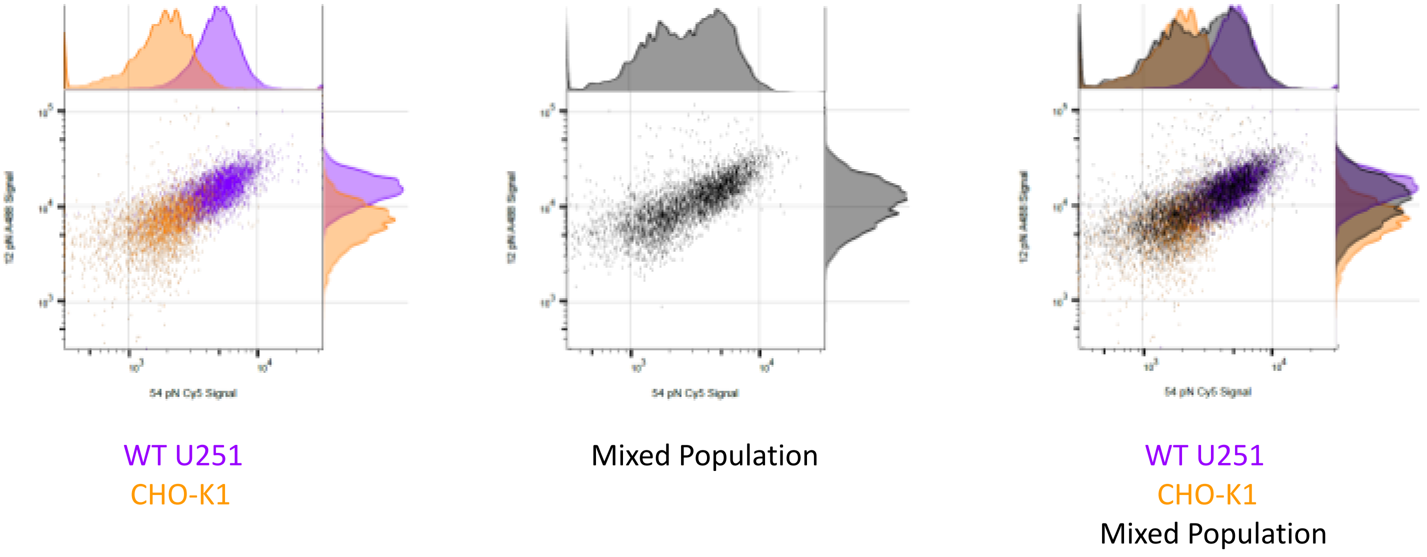

Supplementary Fig. 17. Multiplex RAD-TGTs with inverted fluorophores. a. Scatter plots of U251 and CHO-K1 cells plated on surfaces containing a 12 pN A488 labelled RAD-TGT and 54 pN Cy5 labelled RAD-TGT for both each cell individually and in a mixed population. The fluorophores and TGTs are inverted compared to main figure 3d. Source data are provided as a Source Data file.

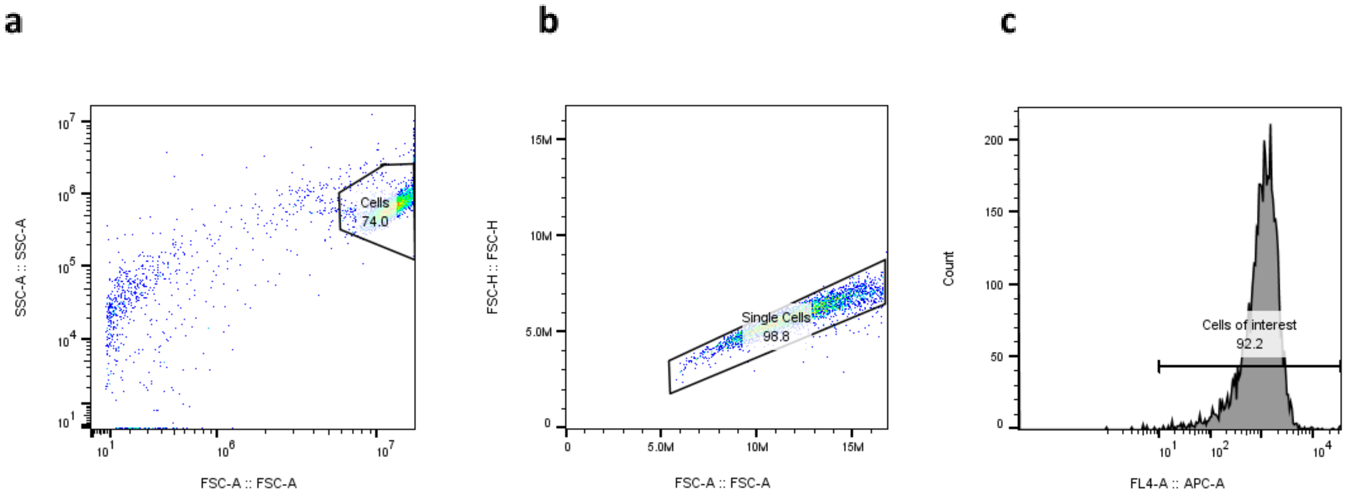

Supplementary Fig. 18. Gating strategy. a. Population of cells was first identified by creating a forward vs side scatter Logicle plot. **b**. Population identified in a was then then gated to isolate single cells only, this was done by graphing the forward scatter area by height and gating for cells that display a linear relationship. **c**. Cells from b are then gated to remove any non fluorescent data points. Cells were displayed as a histogram of the fluorescent intensity of the fluorophore of interest and any point with an intensity greater than 10 were collected.

Supplementary Table 1:

Year	Paper	Cell line tested	Key Findings Related to our Manuscript	Alterations from Original TGT Assay
2013	Defining single molecular forces required to activate integrin and notch signaling.[1]	CHO-K1 and others	First TGT study, demonstrated that cells required a minimum tension tolerance of 43 pN to adhere to TGTs with an RGD ligand	
2015	Integrin Molecular Tension within Motile Focal Adhesions.[2]	CHO-K1	Identified that streaking rupture pattern is caused by motile focal adhesions Utilizes Biotin-BSA coating of surfaces	Utilizes biotin-BSA coating of surfaces
2016	Mechanically Induced Catalytic Amplification Reaction for Readout of Receptor-Mediated Cellular Forces[3]	3t3 and mouse platelets	First non-microscopy based analysis of TGT rupture, amplification of ruptured anchor strand was readout in a plate reader. Quantified total rupture per well	Used an isothermal amplification technique of the exposed anchor strand to generate a robust signal detectable by plate reader.
2016	Constructing Modular And Universal Single Molecule Tension Sensor Using Protein G To Study Mechano-Sensitive Receptors.[4]	DLD1 cells	Utilized Protein G as the ligand, this inturn conjugates to antibodies thus offering a wider array of ligands	Used Protein G -Antibody for the ligand instead of RGD
2018	Integrins Outside Focal Adhesions Transmit Tensions During Stable Cell Adhesion.[5]	CHO-K1	Blebbistatin decreases both 12 and 54 pN rupture when cells are able to fully adhere, much like what is seen with the echi ligands.Very modest decrease in 12 pN rupture.	Both 12 and 54 pN TGTs present on surface
2021	Imaging Integrin Tension and Cellular Force at Submicron Resolution with an Integrative Tension Sensor [6]	Dog platelets	Integrative tension sensors (ITS) (a DNA duplex based tension sensor) were functional when coplated with fibronectin. Researchers used ITS to measure the cumulative rupture the cell generates.	Fibronectin was plated with the tension sensors and cumulative rupture was measured.
2021	Mechanically Triggered Hybridization Chain Reaction.[7]	3t3 and mouse platelets	A further improvement on the plate reader based TGT assay that demonstrates the applications of high throughput mechanotyping by characterizing the Mechano-ic50	Used a hybriziation chain reaction to further (and rapidly) improve TGT rupture signal in a plate reader
2021	DNA-Based Microparticle Tension Sensors (μTS) for Measuring Cell Mechanics in Non-planar Geometries and for High-Throughput Quantification. [8]	Platelets	Silica beads were coated with TGTs that would fluoresce upon rupture and beads were then analyzed. This is the fist flow cytometry based TGT assay but does not provide information on what cell caused the mechanical event	TGTs were fixed to silica beads which allowed for a flow cytometry based readout of the beads and the geometry of the bead allows for TGTs to access constrained regions of cells
2022	Single-Molecule Force Imaging Reveals That Podosome Formation Requires No Extracellular Integrin-Ligand Tensions or Interactions. [9]	THP-1	Unzipping conformation of a DNA-PNA TGT with an RGD ligand saw significant decrease in a TGT rupture	Developed a nuclease resistant TGT using a PNA-DNA duplex to probe invadosome biology
2022	Single-molecule characterization of subtype-specific β1 integrin mechanics [10]	BJ5ta	LDVP ligand allowed analysis of the α4β1 integrin exclusively, this subtype had different mechanical properties such as minimum force requirment and what forces are altered by myosin inhibition	This is the first time a leukocyte specifc integrin was analyzed via TGTs. LDVP was used in lieu of RGD as ligand allowing for different integrin subtypes to be explored

Supplementary Table 1. Prior studies that utilized TGTs and or further improved upon design. This is not a comprehensive list but is representative of recent advances that paralleled our work.

Supplementary References

1. Wang, X. & Ha, T. Defining single molecular forces required to activate integrin and notch signaling. *Science* **340**, 991–994 (2013).
2. Wang, X. *et al.* Integrin Molecular Tension within Motile Focal Adhesions. *Biophys. J.* **109**, 2259–2267 (2015).
3. Ma, V. P.-Y. *et al.* Mechanically Induced Catalytic Amplification Reaction for Readout of Receptor-Mediated Cellular Forces. *Angew. Chem. Int. Ed Engl.* **55**, 5488–5492 (2016).
4. Wang, X. *et al.* Constructing modular and universal single molecule tension sensor using protein G to study mechano-sensitive receptors. *Sci. Rep.* **6**, 21584 (2016).
5. Wang, Y. & Wang, X. Integrins outside focal adhesions transmit tensions during stable cell adhesion. *Sci. Rep.* **6**, 36959 (2016).
6. Zhao, Y., Wetter, N. M. & Wang, X. Imaging Integrin Tension and Cellular Force at Submicron Resolution with an Integrative Tension Sensor. *J. Vis. Exp.* (2019) doi:10.3791/59476.
7. Duan, Y. *et al.* Mechanically Triggered Hybridization Chain Reaction. *Angew. Chem. Int. Ed Engl.* **60**, 19974–19981 (2021).
8. Hu, Y. *et al.* DNA-Based Microparticle Tension Sensors (μTS) for Measuring Cell Mechanics in Non-planar Geometries and for High-Throughput Quantification. *Angew. Chem. Int. Ed Engl.* **60**, 18044–18050 (2021).
9. Pal, K., Tu, Y. & Wang, X. Single-Molecule Force Imaging Reveals That Podosome Formation Requires No Extracellular Integrin-Ligand Tensions or Interactions. *ACS Nano* **16**, 2481–2493 (2022).
10. Jo, M. H. *et al.* Single-molecule characterization of subtype-specific β1 integrin mechanics. *Nat. Commun.* **13**, 7471 (2022).
