# Supplementary material for: RAD-TGTs: high-throughput measurement of cellular mechanotype via rupture and delivery of DNA tension probes
Source: Nat Commun. 2023 Apr 28;14:2468. doi: 10.1038/s41467-023-38157-6 (PMC10147940; doi:10.1038/s41467-023-38157-6)
Supplement: Supplementary file 3 — Reporting Summary [file 41467_2023_38157_MOESM3_ESM.pdf]

nature portfolio

Corresponding author(s): Wendy R. Gordon

Last updated by author(s): Apr 3, 2023

Reporting Summary

Nature Portfolio wishes to improve the reproducibility of the work that we publish. This form provides structure for consistency and transparency in reporting. For further information on Nature Portfolio policies, see our Editorial Policies and the Editorial Policy Checklist.

Statistics

For all statistical analyses, confirm that the following items are present in the figure legend, table legend, main text, or Methods section.

n/a	Confirmed	
☐	☒	The exact sample size (*n*) for each experimental group/condition, given as a discrete number and unit of measurement
☐	☒	A statement on whether measurements were taken from distinct samples or whether the same sample was measured repeatedly
☐	☒	The statistical test(s) used AND whether they are one- or two-sided<br>*Only common tests should be described solely by name; describe more complex techniques in the Methods section.*
☒	☐	A description of all covariates tested
☐	☒	A description of any assumptions or corrections, such as tests of normality and adjustment for multiple comparisons
☐	☒	A full description of the statistical parameters including central tendency (e.g. means) or other basic estimates (e.g. regression coefficient) AND variation (e.g. standard deviation) or associated estimates of uncertainty (e.g. confidence intervals)
☐	☒	For null hypothesis testing, the test statistic (e.g. *F*, *t*, *r*) with confidence intervals, effect sizes, degrees of freedom and *P* value noted<br>*Give P values as exact values whenever suitable.*
☒	☐	For Bayesian analysis, information on the choice of priors and Markov chain Monte Carlo settings
☒	☐	For hierarchical and complex designs, identification of the appropriate level for tests and full reporting of outcomes
☒	☐	Estimates of effect sizes (e.g. Cohen's *d*, Pearson's *r*), indicating how they were calculated

Our web collection on statistics for biologists contains articles on many of the points above.

Software and code

Policy information about availability of computer code

Data collection	-All flow cytometry data collected with the BD Accuri C6 Plus (64-bit) Software Version 1.0.34.1<br>- Images collected with EVOS FL Auto Software Revision 1.7
Data analysis	- Flow cytometry data was gated and analyzed with FlowJo Vl0.8<br>- Statistical analysis and superplots were generated in Graph Pad Prism 9<br>- Images were processed in ImageJ l.53q with the FIJI package<br>-EditR was used to quantify sanger sequencing software found at http://baseeditr.com/<br>-Next generation sequencing was analyxed with custom code at https://github.com/AdamTSmiley/TGT_Barcode_Counter

For manuscripts utilizing custom algorithms or software that are central to the research but not yet described in published literature, software must be made available to editors and reviewers. We strongly encourage code deposition in a community repository (e.g. GitHub). See the Nature Portfolio guidelines for submitting code & software for further information.

Data

Policy information about availability of data

All manuscripts must include a data availability statement. This statement should provide the following information, where applicable:

- Accession codes, unique identifiers, or web links for publicly available datasets
- A description of any restrictions on data availability
- For clinical datasets or third party data, please ensure that the statement adheres to our policy

Source data are provided with this paper. Further data that supports this work is available from the corresponding author upon reasonable request. The raw data from barcoding experiments is provided with the code in the "Code Availability" section.

Human research participants

Policy information about studies involving human research participants and Sex and Gender in Research.

Reporting on sex and gender	N/A
Population characteristics	N/A
Recruitment	N/A
Ethics oversight	N/A

Note that full information on the approval of the study protocol must also be provided in the manuscript.

Field-specific reporting

Please select the one below that is the best fit for your research. If you are not sure, read the appropriate sections before making your selection.

☒ Life sciences ☐ Behavioural & social sciences ☐ Ecological, evolutionary & environmental sciences

For a reference copy of the document with all sections, see nature.com/documents/nr-reporting-summary-flat.pdf

Life sciences study design

All studies must disclose on these points even when the disclosure is negative.

Sample size	The amount of cells plated for each experiment was measured using the Thermo Fischer Countess Ill, amount of cells plated were chosen based on cell confluency on a 96 well plate.we demonstrated by testing a gradient of cell counts that changing the number of cells plated has little to no effect on signal.<br>Experiments were performed in triplicate to allow for robust statistical analysis<br>The number of cells analyzed were automatically counted by the BD Accuri C6 Plus personal cytometer.
Data exclusions	No data were excluded, except during flow cytometry gating, which gated out cell debris, dead cells, and cell clusters. Furthermore, events with 0 fluorescence were excluded this appeared to be an artefact from the cytometer, even in cases of no fluorophore present cells had an intrinsic fluorescent value and on random dates also had some 0value events. We concluded due to the cells intrinsic fluorescence value that the 0 values were not cells
Replication	All experiments unless otherwise noted were completed in triplicate with each replicate performed on a separate day, all attempts were successful
Randomization	Cells that were subjected to any treatment were selected randomly and control cells derived from the same plate as experimental cells. Otherwise no further randomization was performed.
Blinding	Blinding was not possible to ensure the sample received correct treatment and were indeed the sample of interest

Reporting for specific materials, systems and methods

We require information from authors about some types of materials, experimental systems and methods used in many studies. Here, indicate whether each material, system or method listed is relevant to your study. If you are not sure if a list item applies to your research, read the appropriate section before selecting a response.

Materials & experimental systems

n/a	Involved in the study
☒	☐ Antibodies
☐	☒ Eukaryotic cell lines
☒	☐ Palaeontology and archaeology
☒	☐ Animals and other organisms
☒	☐ Clinical data
☒	☐ Dual use research of concern

Methods

n/a	Involved in the study
☒	☐ ChIP-seq
☐	☒ Flow cytometry
☒	☐ MRI-based neuroimaging

Eukaryotic cell lines

Policy information about cell lines and Sex and Gender in Research

Cell line source(s)	CHO-Kl cells provided from ATCC<br>U251 WT and KOs were provided from the Odde and Largaespada Labs, WT was originally obtained from G. Y. Gillespie (The University of Alabama at Birmingham)
Authentication	No authentication was performed for cells directly provided by ATCC; U251 cell lines were authenticated with SSLP analysis (https://uagc.arl.arizona.edu/services/complete-solutions/cell-line-authentication) and knockouts were confirmed with Western blots.
Mycoplasma contamination	CHO-K1, U251, U251-talinKO, and U251-CD44KO were tested for mycoplasma every 3 months. All cells tested negative for contamination
Commonly misidentified lines<br>(See ICLAC register)	none

Flow Cytometry

Plots

Confirm that:

☒ The axis labels state the marker and fluorochrome used (e.g. CD4-FITC).

☒ The axis scales are clearly visible. Include numbers along axes only for bottom left plot of group (a 'group' is an analysis of identical markers).

☒ All plots are contour plots with outliers or pseudocolor plots.

☒ A numerical value for number of cells or percentage (with statistics) is provided.

Methodology

Sample preparation	Cells were plated at the densities as specified in the main text and were subjected to described treatment. Following approriate incubation media was removed from the cells and the cells were released with Cell lines were seeded at densities and in plates indicated in the paper 1 day prior to the experiment. After incubation with the material of interest, they were washed in phosphate buffered saline (PBS) and detached with TrypLE Express Enzyme for 5 minutes at 37 Celcius. Cells were resuspended in a PBS solution containing 1% bovine serum albumin and 1 mM EDTA, cells were then analyzed via flow cytometry
Instrument	BD Accuri C6 Plus Personal Flow Cytometer
Software	BD Accuri C6 Plus (64-bit) Software version 1.034.1
Cell population abundance	All cell lines were homogeneous, containing only one population
Gating strategy	Cell populations were selected by finding the population in a forward vs side scatter logicle plot followed gating for single cells by selecting for cells that maintianed a linear relation ship between area and height forward scatter. Following this cells that contained non zero fluorescence were selected see data exclusion for reasoning

☒ Tick this box to confirm that a figure exemplifying the gating strategy is provided in the Supplementary Information.
