# Supplementary figures and images for: RAD-TGTs: high-throughput measurement of cellular mechanotype via rupture and delivery of DNA tension probes
Source: Nat Commun. 2023 Apr 28;14:2468. doi: 10.1038/s41467-023-38157-6 (PMC10147940; doi:10.1038/s41467-023-38157-6)

## Slide 1
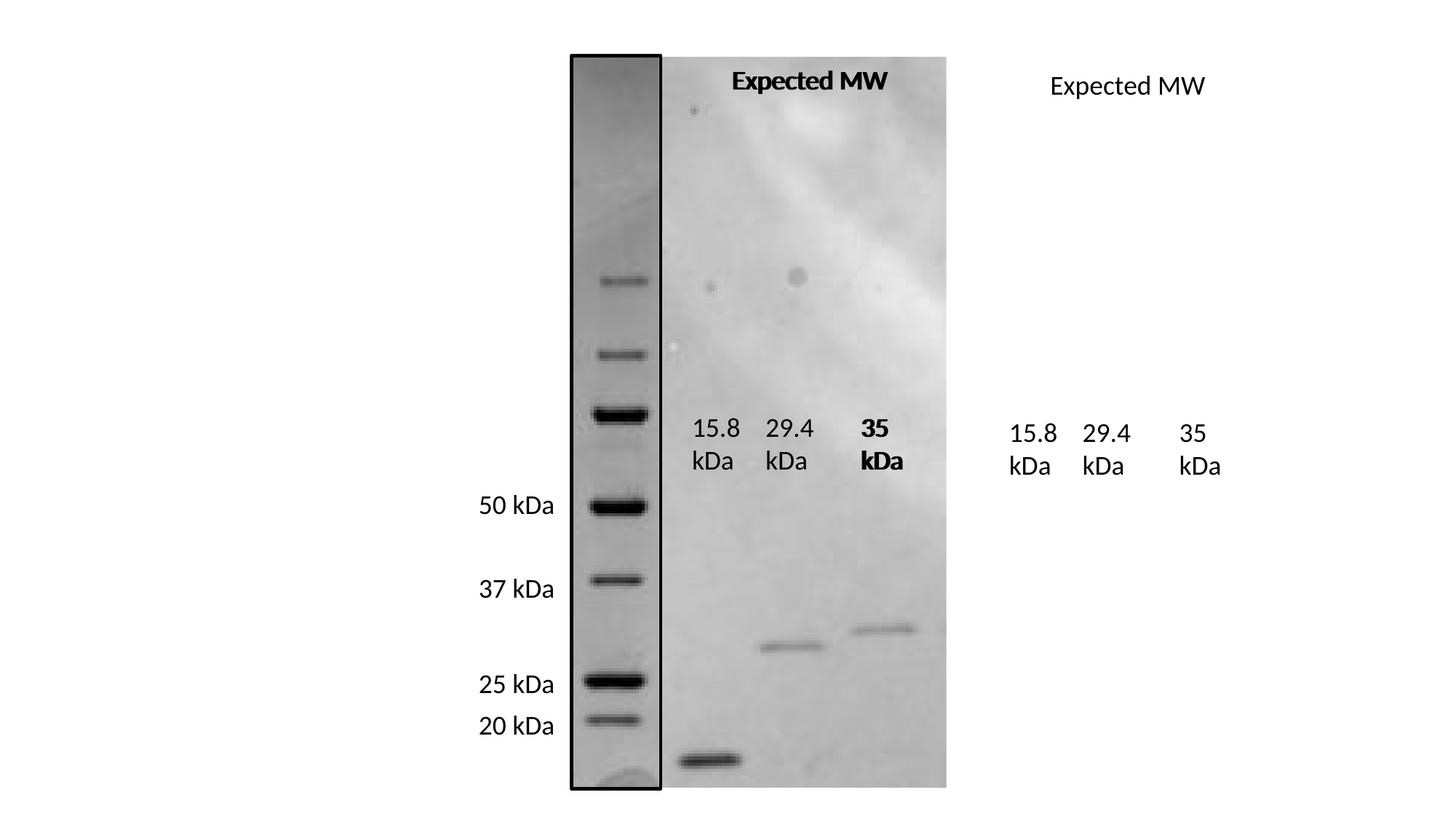

Expected MW
Expected MW
Expected MW
35 kDa
35 kDa
29.4 kDa
15.8 kDa
35 kDa
29.4 kDa
15.8 kDa
50 kDa
37 kDa
25 kDa
20 kDa

## Slide 2
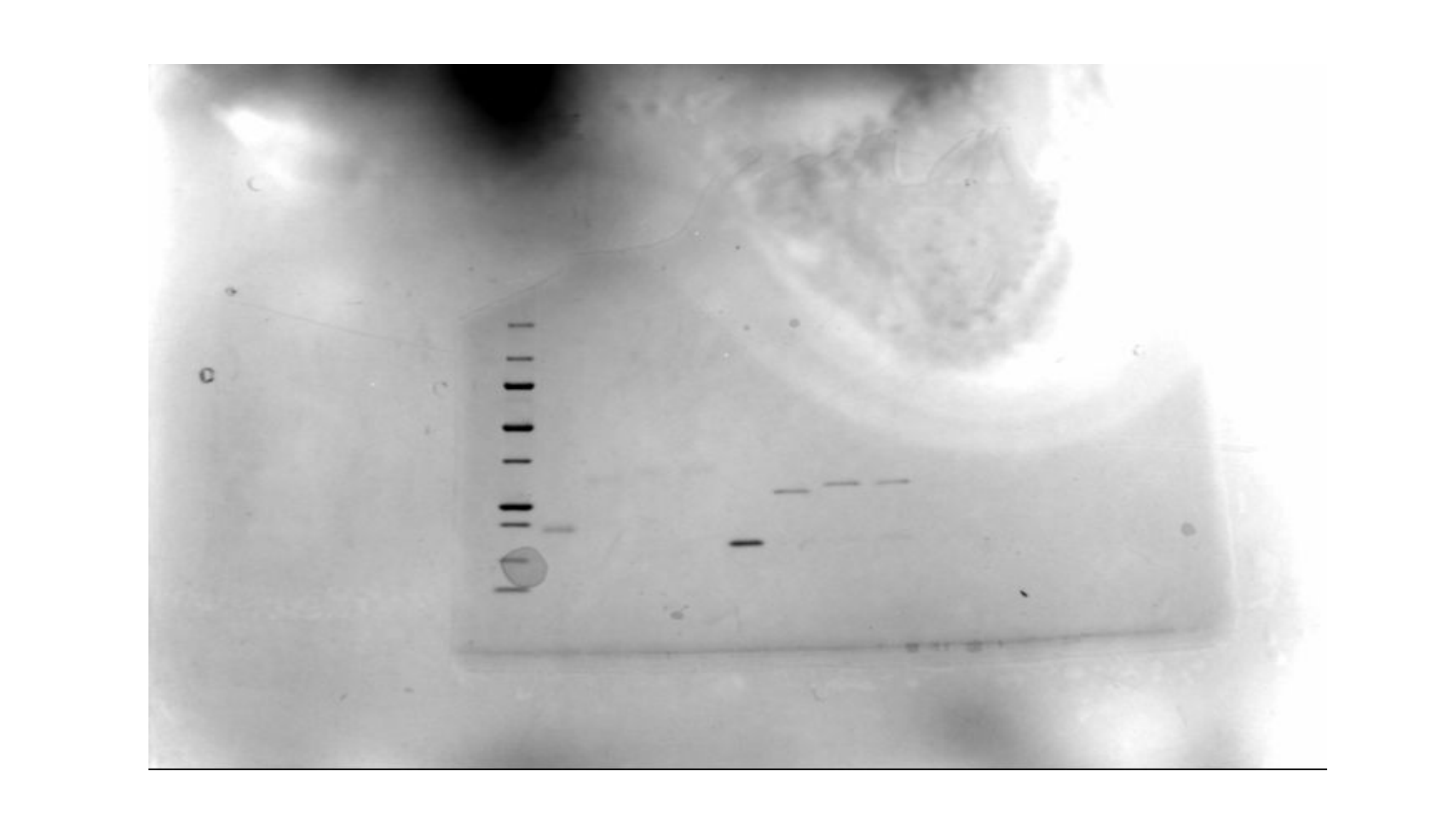

Supplement: Supplementary file 4 — Source data [file 41467_2023_38157_MOESM4_ESM.zip › Source Files/Non-excel Source Files/Source file sup 2.pptx]
